# Understanding the Genetic Domestication History of the Jianchang Duck by Genotyping and Sequencing of Genomic Genes Under Selection

**DOI:** 10.1534/g3.119.400893

**Published:** 2020-03-12

**Authors:** Lei Wang, Jiazhong Guo, Yang Xi, Shengchao Ma, Yanying Li, Hua He, Jiwen Wang, Chunchun Han, Lili Bai, Ahsan Mustafa, Hehe Liu, Liang Li

**Affiliations:** * Farm Animal Genetic Resources Exploration and Innovation Key Laboratory of Sichuan Province, College of Animal Science and Technology, Sichuan Agricultural University, Chengdu, P.R. China; † Institute of Animal Nutrition, Key Laboratory for Animal Disease-Resistance Nutrition of China, Ministry of Education, Sichuan Agricultural University, Chengdu, P.R. China

**Keywords:** Jianchang duck, domestication, molecular phylogeny, selection signature, plumage coloration

## Abstract

The Jianchang duck is mainly distributed in Southwest China, and has the characteristics of fast growth rate and strong abilities in lipid deposition in the liver. In order to investigate the effects of domestication process on formation of the unique characteristics of Jianchang duck, the whole genome of sixteen individuals and three pooling of Jianchang duck were re-sequenced, and genome data of 70 mallards and 83 domestic ducks from thirteen different places in China were obtained from NCBI. The population stratification and evolution analysis showed gene exchanges existed between the Jianchang and other domestic duck populations, as well as Jianchang ducks and mallards. Genomic comparison between mallards and Jianchang ducks showed genes, including *CNTN1*, *CHRNA9*, and *SHANK2*, which is involved in brain and nerve development, experienced strong positive selection in the process of Jianchang duck domestication. The genomic comparison between Jianchang and domestic duck populations showed that *HSD17B12* and *ESM1*, which affect lipid metabolism, experienced strong positive selection during the domestication process. *F*_ST_ analysis among populations of Jianchang duck with different plumage colors indicated that *MITF* was related to the phenotype of a white feather, while *MC1R* was related to the phenotype of hemp feather. Our results provided a base for the domestication process of Jianchang duck and the genomic genes for unique traits.

Domestic ducks are domesticated from mallards since about 2,200 years ago in China as a single domestication event (Qu *et al.* 2009; Zhang *et al.* 2018). Under the impacts of directional artificial selection, the ducks were classified into three types depending on the usage purpose, including egg type, meat type, and dual-purpose type (Su *et al.* 2007). During the domestication process of duck genes that affect the brain and neuron development, which undergone a strong positive selection effect (Zhang *et al.* 2018). And the genes that refer to plumage color were selected as well, *e.g.*, the alleles of *MITF* genotypes were already fixed in Pekin duck, a famous white plumage meat-type duck breed (Zhou *et al.* 2018).

Jianchang ducks, mainly distributed in Southwest China (Liangshan, Sichuan province), have four populations according to their feather color phenotypes, including white feather, light hemp feather, deep hemp feather and white chest black feather (Figure 1A-D) (Yang *et al.* 1996). Compared with other local duck breeds in China, Jianchang ducks grow fast and easily deposit lipid in the liver (Jiansheng 1987; Wang *et al.* 2018). These characteristics may be related to natural living environments and long-term artificial selection. The natural environment and geographical isolation in Southwest China, have led to genetic changes of many species (Li *et al.* 2013; Wang *et al.* 2015; Lan *et al.* 2018). Particularly, the high altitude and low temperature in Liangshan areas (Liu *et al.* 2017) may improve the ability of Jianchang duck to resist the cold temperature through increasing fat deposition and body size. Besides that, the Hui nationality people living in Liangshan area for hundreds of years (Kongshao 2005), prefer to take the belly fat of stuffed salted duck as cooking oil, which may also lead to an indirect selection on the enhancement of fat deposition ability of Jianchang duck.

**Figure 1:**
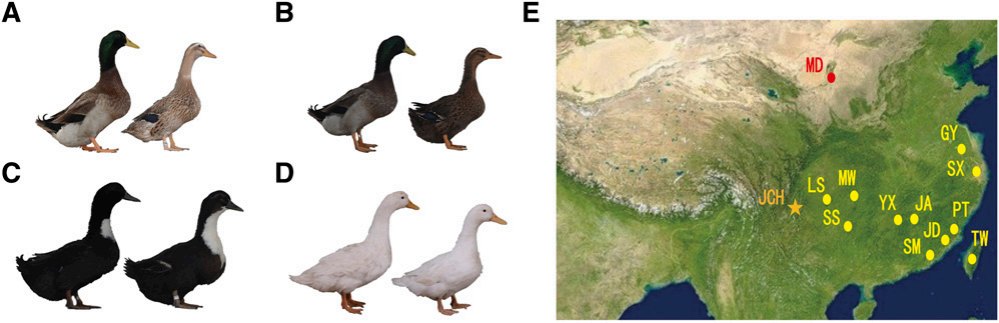
Graphical representation of Jianchang ducks and geographical distribution of the selected ducks in this study. **(A)** Light hemp feather population **(B)** deep hemp feather population **(C)** white chest and black feather population **(D)** white feather population **(E)** the geographical distribution of duck population. Orange star represent the distribution areas for Jianchang duck, red dots represent the distribution areas for mallards, and yellow dots represent living areas for other domesticated duck.

The long-term domestication process will produce genetic footprints on the genomes of animals (Frantz and Larson 2018; Guo *et al.* 2018; Wilkinson and Wiener 2018). We suspected that there would be genetic changes underlying formation of the characteristics of Jianchang duck during long-term domestication process. Analysis of microsatellite and mtDNA markers demonstrated that the genetic background of Jianchang duck was significantly different from other local breeds (Qu *et al.* 2009). In order to investigate the effects of domestication process on formation of the unique characteristics of Jianchang duck, it is necessary to further investigate the genetic footprints of Jianchang duck at a nuclear genome view basing on genome re-sequencing. These analyses could provide a basis for further evaluation of the unique genetic foundation of Jianchang duck.

## Materials and Methods

### Sampling and DNA isolation

All the experimental procedures, described below, were approved by the Animal Ethics Monitoring Committee of Sichuan Agriculture University and carried out in accordance with Guideline of Animal Welfare China. Whole blood samples were taken from the duck wing vein using standard venipuncture, and genomic DNA was extracted using standard phenol/chloroform extraction method. Subsequently, the concentration and purity of the extracted DNA were detected using an ultraviolet spectrophotometer. In individual sequencing, sixteen Jianchang duck individuals were selected. Total sixteen individuals were selected from the four populations for resequencing. *i.e.*, deep hemp feather (n = 4), shallow hemp feather (n = 4), white feather (n = 4), and white chest black feather (n = 4). Each feather color population includes half male and half female. In pooling sequencing, total three pooling DNA were prepared and each pooling was mixed with DNA from twenty individuals, definitely, hemp feather pooling includes ten deep hemp and ten shallow hemp individuals, white feather pooling includes twenty white feathers individuals, white chest black feather pooling includes twenty white chest black feathers individuals. Each pooling includes half males and half females, and a similar amount of DNA for each individual was mixed (Supplementary Tables S1). The total DNA concentration was above 100ng / ul, and the total DNA content was above 20 ug.

### Library preparation, high-throughput sequencing and download sequenced data

For each sample, two paired-end libraries (150 bp) were constructed according to the manufacturer protocols (Illumina) and sequenced on the Illumina Hiseq 2500 sequencing platform. The sixteen individuals were sequenced at 5**×** coverage, and the three pooling DNA samples were sequenced at 20**×** coverage (Supplementary Tables S2).

Clean data of genome 153 duck individuals from thirteen places in China were downloaded from NCBI (https://www.ncbi.nlm.nih.gov) and their information was provided in supplementary data (Supplementary Tables S3). These data were included Banzui duck (BZ; n = 2), Longsheng duck (LS; n = 2), Mawang duck (MW; n = 2), Sansui duck (SS; n = 2), Putian duck (PT; n = 2), Ji’an duck (GA; n = 2), Taiwan duck (TW; n = 2), Youxian duck (YX; n = 2), Gaoyou duck (GY; n = 10), Jinding duck (JD; n = 10), Shanma duck (SM; n = 10), Shaoxing duck (SX; n = 37) and Mallard (MD; n = 70).

### Sequence filtering, read alignment and variation detection

The raw reads were filtered using NGS QC (v2.3.3) Toolkit (Corkins *et al.* 2017) with default parameters. The filtered reads were mapped to the *Anas platyrhynchos* genome (duckbase.refseq.v4; (Yu *et al.* 2018) using BWA (v0.7.12;(Jo and Koh 2015) with default parameters. After aligning the reads, SNPs and small indels (1-50 bp) were called used GATK (v4.0.11.0; (Nielsen *et al.* 2011) with the parameter of minimum quality score of 20 on the mapping result. Finally, all SNPs were retained and filtered using GATK with including parameters: -T SelectVariants -selectType SNP -select ” AF < 0.95 “, and high-quality SNPs were retained in the form of V*CF*.

### Population stratification analysis

Neighbor-joining phylogenetic tree was then built using the snphylo (v2.0) with default parameters through the VCF file (Lee *et al.* 2014). And the iTOL website (http://itol.embl.de/index.shtml) was used to beautify the phylogenetic tree (Chen *et al.* 2018).

GCTA (v1.25) was used for Principle Component Analysis (PCA) to extract the genetic relationship matrix of the first four eigenvectors generated with default parameters (Yang *et al.* 2011; Slifer 2018). Then admixture (v1.3.0) was used to analyze the population genetic structure (Peter 2016). Presume ancestral populations (K) between two and six were run with 10,000 iterations.

### Population evolutionary analysis

Treemix (v1.13) with default parameters except for the “-root BZ -k 500 -m 3” option was used for gene flow analysis (Klimova *et al.* 2018). Pairwise Sequentially Markovian Coalescence (PSMC) model was used to infer population history dynamics though bam files by mapping (Nadachowska-Brzyska *et al.* 2015). The history of population size over time was reconstructed by applying the distribution of the nearest common ancestor between the two alleles in the sample. Parameters of PSMC analysis are set as “-n25-t15-r5-p “4+25*2+4+6” and “-g 1-u 0.2e-08”, respectively.

### Selective sweep and functional enrichment analyses

In order to define the selection characteristics associated with Jianchang duck and the candidate regions for the targeted selection of Jianchang duck during domestication, we used Vcftools (v0.1.13) to calculate the fixation statistics (*F*_ST_) and population nucleotide diversity ratio θπ (other / Jianchang) and θπ (wild / Jianchang) (Liu *et al.* 2018 *preprint*), and two complementary methods were used to identify areas that may be affected by long-term selection. The average *F*_ST_ and θπ were calculated in 20kb windows with a 10kb shift. The logarithmic function was used to transform the θπ ratio. The *F*_ST_ and θπ logarithmic advantage ratio of the top 5% of windows were considered as putative selection target regions. The resulting sites were genetically annotated by Bedtools (v2.17.0; (Quinlan and Hall 2010), and the selected genes were subjected to GO analysis and KEGG analysis using DAVID (v6.8) and KOBAS (v3.0) to annotate the function of the selected genes (Yang *et al.* 2018).

### Pooling data for F_ST_ analysis

Popoolation2 (Kofler *et al.* 2011) was used to calculate *F*_ST_ values between different feather color pooling VCF files after filtering. The *F*_ST_ values were averaged over SNPs in a 5 kb sliding window with a 2.5 kb step size for each comparison groups.

### Data availability

The raw data files obtained in this study by HiSeq XTen sequencing have been submitted to the Sequence Read Archive Database of the National Center for Biotechnology Information. The BioProject accession number is PRJNA548668 and the BioSample accession numbers of sequence read archive runs of sixteen samples are SAMN12046286, SAMN12046287, SAMN12046288, SAMN12046289, SAMN12046290, SAMN12046291, SAMN12046292, SAMN12046293, SAMN12046294, SAMN12046295, SAMN12046296, SAMN12046297, SAMN12046298, SAMN12046299, SAMN12046300, SAMN12046301. Genomic data selected from published local breeding genomic data of duck are available in Supplemental Tables S3. Supplemental material available at figshare: https://doi.org/10.25387/g3.11974002.

## Results

### Population structure

**Figure 2:**
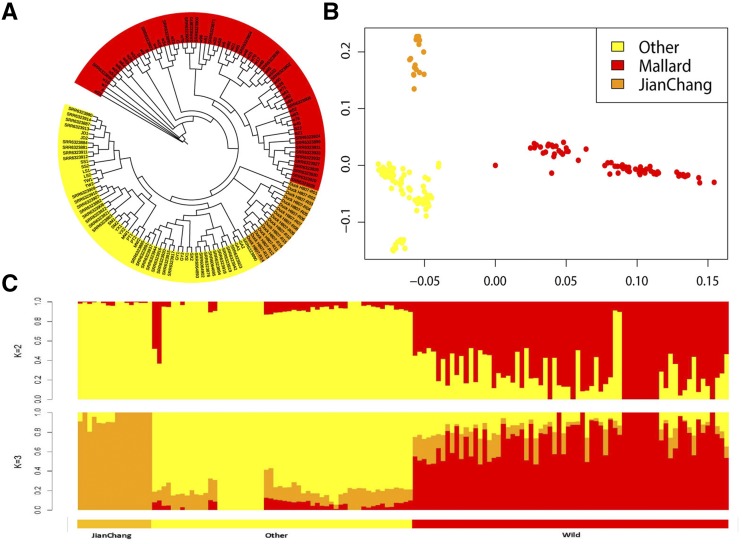
Population stratification analysis of Jianchang duck. **(A)** Neighbor-joining phylogenetic tree of fourteen duck populations. **(B)** Principal Component Analysis (PCA). **(C)** Population structure analysis. Jianchang ducks are marked in orange, other domestic ducks are in yellow, and the mallards are in red.

Genome-wide population stratification analysis was conducted to show the population genetic structure and relationships of Jianchang duck (Figure 1A-D) with other duck populations (Figure 1E). The neighbor-joining (NJ) of pairwise genetic distances based on whole genome-wide SNPs (Figure 2A) showed that Chinese duck breeds can be mainly classified into wild and domesticated groups. From the categories of domesticated ducks, Jianchang duck was present in an independent branch. PCA (Figure 2B) analysis revealed that the whole duck population was divided into three groups: wild, Jianchang and other domesticated. Further population structure analysis (Figure 2C) showed that when K = 2, all individuals were divided into two groups: wild and domesticated ducks; when K = 3, a clear division existed between Jianchang and other domesticated populations in the domesticated population, and the Cross-Validation error is the lowest.

### Gene flow and demographic history analysis

**Figure 3:**
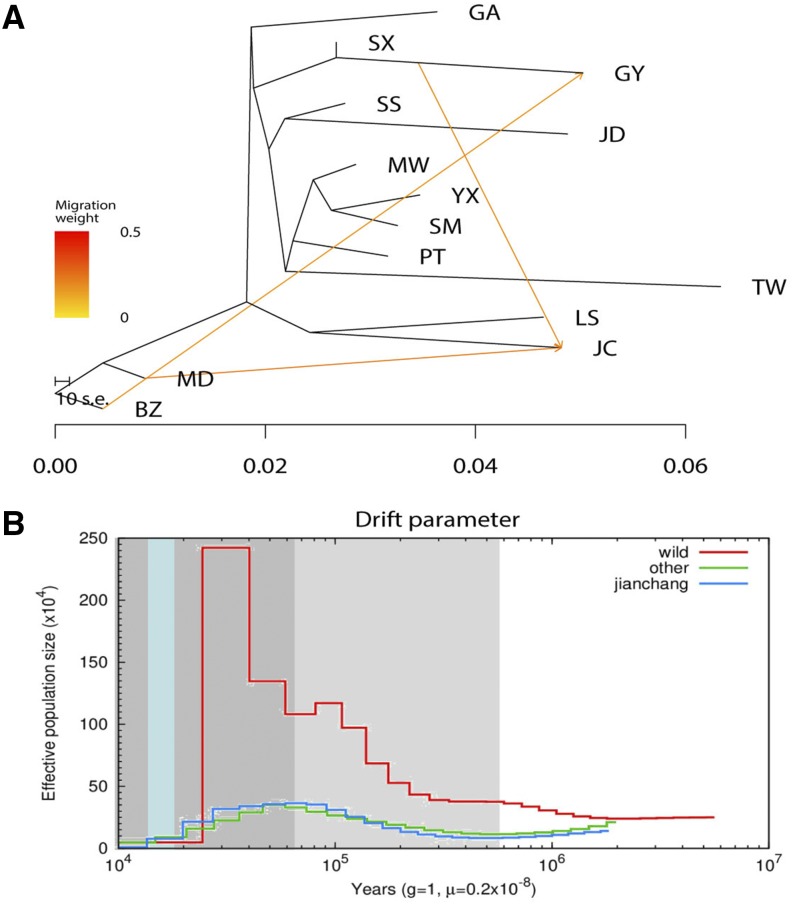
Population evolution analyses of Jianchang duck. **(A)** Gene flow analysis. **(B)** Demographic history of duck population analysis. The line represents the estimated population size, the light gray shaded area represents the Pleistocene period, the last ice age (LGP) is in the dark gray shaded, and the last ice age (LGM) is in light blue. Red, green and blue lines inside the graph represent populations of wild, Jianchang and other domestic duck, respectively.

Gene flow analysis (Figure 3A) showed that Jianchang duck (designed as JC) was the first group of ducks to be separated from domestic ducks, which is the closest relative to Longsheng duck. There were genetic exchanges between the Jianchang and other domestic populations, as well as between Jianchang and wild populations. PSMC analysis showed (Figure 3B) that the development trend of the quantitative kinetics of Jianchang duck population was similar to that of other domestic duck population, but far from that of the wild duck population. The trend of the effective ancestor population size of these three groups increased in the interglacial periods and decreased in the Pleistocene.

### Selective sweep and functional enrichment analyses

**Figure 4:**
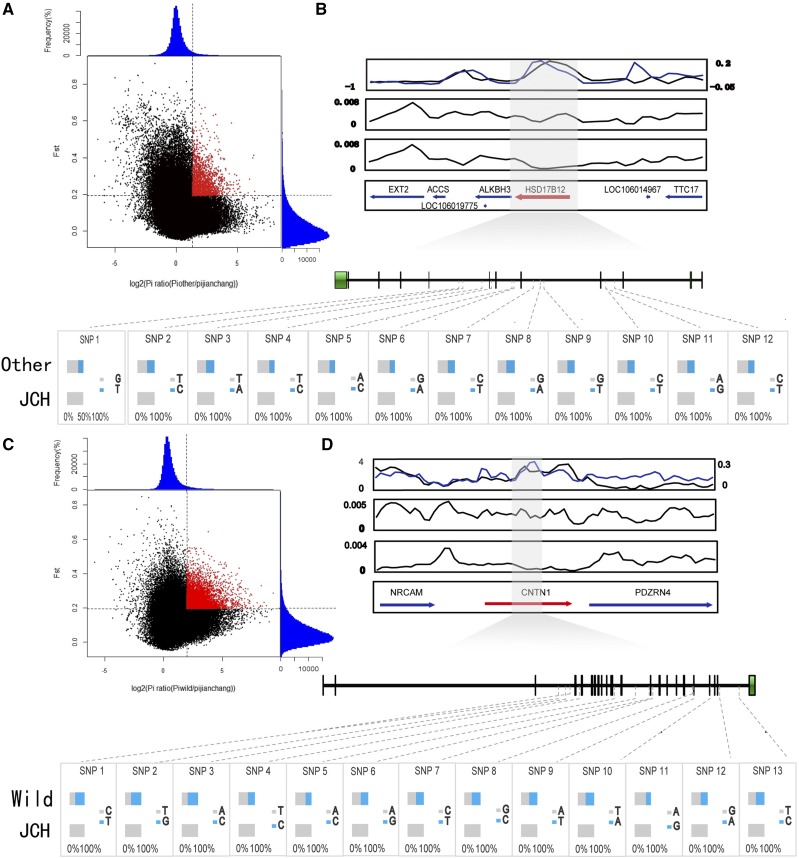
Genomic regions with strong selective sweep signals in Jianchang and wild populations or Jianchang and other domesticated populations. **(A)** Distribution of θπ ratios [θπ (Other domestic ⁄ Jianchang)] and *F*_ST_ values. Data points in red are regions under selection. **(B)** The *HSD17B12* gene showed different genetic signature in domesticated and mallards. The black and red lines represent log_2_ (Other domestic ⁄ Jianchang θπ) ratios and *F*_ST_ values, respectively. The *HSD17B12* gene region is shown in gray. Allele frequencies of twelve SNPs within the *HSD17B12* gene across two duck populations. **(C)** Distribution of θπ ratios [θπ (wild ⁄ Jianchang)] and *F*_ST_ values. Data points in red are regions under selection. **(D)** The *CNTN1* gene showed different genetic signature in Jianchang ducks and mallards. The black and red lines represent log2 (θπ wild ⁄ θπ Jianchang) ratios and *F*_ST_ values, respectively. The *CNTN1* gene region is shown in gray. Allele frequencies of thirteen SNPs within the *CNTN1* gene across two duck populations. Other represents the other domestic ducks, Wild represents the mallards, JCH represents the Jianchang ducks.

The genome-wide variations of Jianchang duck were compared with other domestic duck breeds (BZ, LS, MW, SS, PT, GA, TW, YX, GY, JD, SM, and SX) and mallards, respectively. We filtered all genes located in the top 5% regions with a high *F*_ST_ and pairwise diversity ratio (θπ) among two populations (Supplementary Tables S4). We identified 370 genes in a comparison group of Jianchang and other domestic (Figure 4A, Supplementary Tables S5), and 867 genes in the comparison group of Jianchang and wild (Supplementary Tables S6) (Figure 4C, Supplementary Tables S7).

In the comparison group of Jianchang and other domestic, all 370 genes located in the top 5% *F*_ST_ and θπ regions were used for the GO and KEGG analysis, resulting in a total of 54 GO terms and 76 KEGG enrichment terms. Among the top 10 enriched GO categories (Supplementary Tables S8), three are related to Growth and development, including dystrophin-associated glycoprotein complex, Wnt signaling pathway involved in dorsal/ventral axis specification and coreceptor activity involved in Wnt signaling pathway, and two GO categories are associated with Skeletal phylogeny, including osteoblast development and actin cytoskeleton reorganization. Moreover, two GO categories are associated with Physiological processes, including sialyltransferase activity and peptidase activity. Among the top 10 enriched pathways (Supplementary Tables S9), lipid metabolism displayed the greatest enrichments, followed by nutrition metabolism, with four genes (*ESM1*, *HSD17B12*, PTPRJ and GLI2) showed strong signs of selective sweeps. The *HSD17B12* gene (Figure 4B), which is involved in the biosynthesis of fatty acids (Zhang *et al.* 2017), was located in the prominent position of *F*_ST_ and θπ ratio. Total twelve homozygous or heterozygous SNP sites in five introns of *HSD17B12* were identified in part of other domestic duck individuals, and no plausible SNPs were detected in these sites for the Jianchang duck individuals.

In the comparison group of Jianchang and wild, all 867 genes located in the top 5% *F*_ST_ and θπ regions were used for the GO and KEGG analysis, and finally 121 GO enrichment terms and 119 KEGG enrichment terms were enriched. Among the top 10 enriched categories in GO functional analysis (Supplementary Tables S10), eight GO categories were related to neuromodulation, including receptor complex, positive regulation of cell-substrate adhesion, positive regulation of cell migration, response to endoplasmic reticulum stress, positive regulation of positive chemotaxis, dopamine metabolic process, transmembrane receptor protein tyrosine kinase activity and positive regulation of actin filament polymerization. Among the top 10 KEGG pathways (Supplementary Tables S11), neural related processes displayed the greatest enrichments, and in neural-related processes three genes (*CNTN1*, *CHRNA9*, and *SHANK2*) showed strong signals of selective sweeps. The *CNTN1* gene (Figure 4D) was located in a significant position in *F*_ST_ and θπ ratio, which is involved in neural processes of cerebellar development and retinal axon regeneration (Haenisch *et al.* 2005). Total thirteen homozygous or heterozygous SNP sites in nine introns of *CNTN1* were identified in part of mallards, and no plausible SNPs were detected in these sites for the Jianchang duck individuals.

### Genes related to feather color in Jianchang duck populations

**Figure 5:**
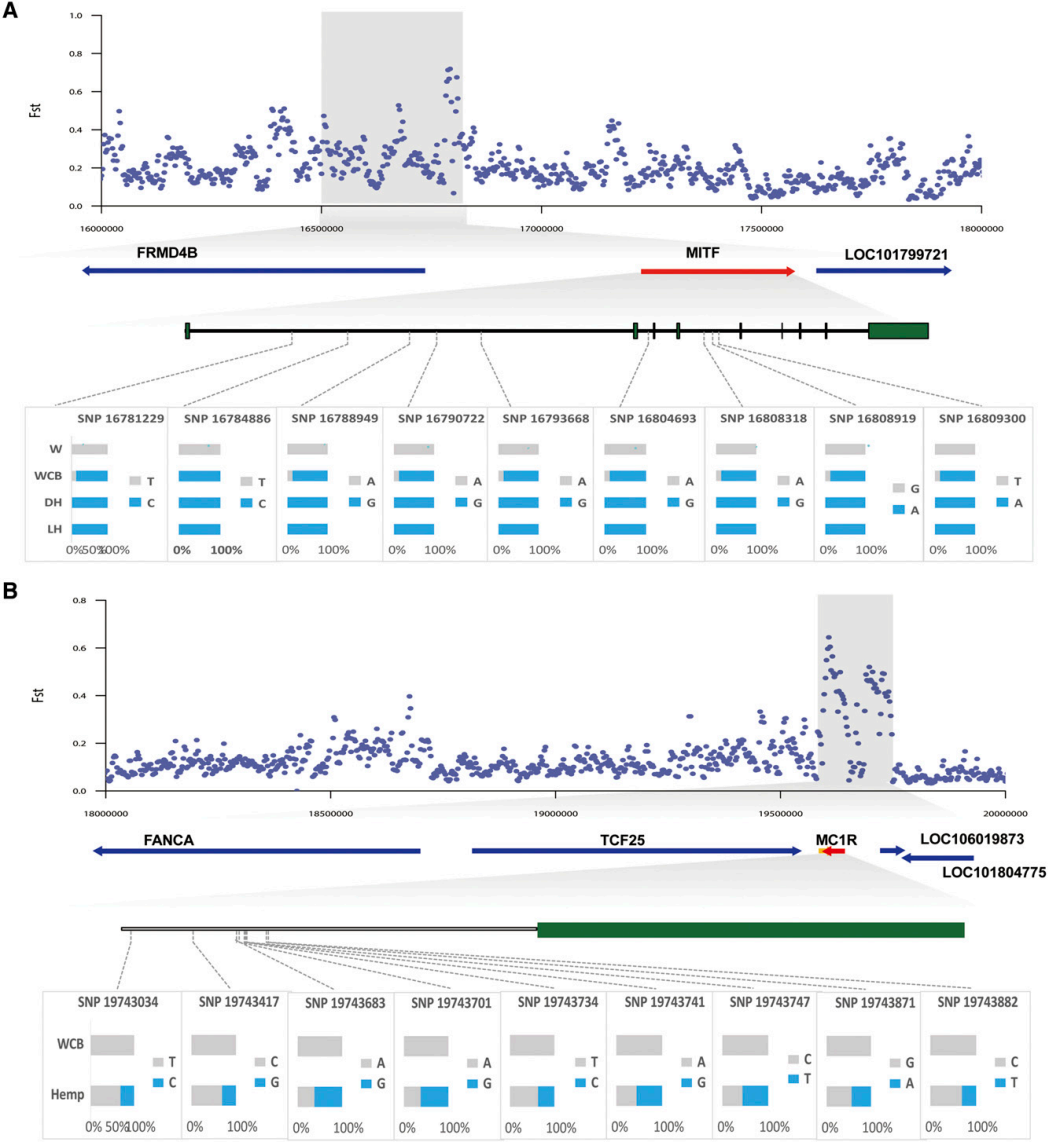
*MITF* and *MC1R* show different genetic signature between different plumage color ducks. **(A)** *F*_ST_ plot around the *MITF* locus. The *MITF* gene is in the shaded area. **(B)** *F*_ST_ plot around the *MC1R* locus. The *MC1R* gene is in the shaded area. SNPs were named according to their position on the scaffold. Blue represents the mutation site and gray represents the reference genome. WCB represents the white chest black plumage ducks, W represents the white plumage ducks, DH represents the deep hemp plumage ducks. SH represents the shallow hemp plumage ducks and Hemp represents the hemp plumage ducks.

When all colored plumage duck populations were compared with the white ones, it was found that one significant selection signals existed on chromosome 13 (Supplementary Figure 5, Figure 5A), whereas, there was no signal when it was compared among the colored plumage duck groups. Further analysis showed that the candidate chromosomal segment under the significant signals on chromosome 13 harbored one gene encompassing *MITF* (*F*_ST_ = 0.72). Moreover, in the intronic region of the *MITF* gene, we identified nine homozygous SNP sites which have already fixed in hemp plumage breeds (n = 8) and nine homozygous or heterozygous SNP sites present in white chest black plumage breeds (n = 4), and these sites in white plumage breeds (n = 4) were consistent with the reference genome (Supplementary Figure 5, Figure 5A). When comparing colored plumage ducks of the white chest black plumage ducks with the hemp ones, we also observed one significant signal existed on chromosome 12 (Figure 5B). Further analysis showed that *MC1R* was distributed in the candidate chromosomal region (*F*_ST_ = 0.64). And nine homozygous or heterozygous SNP sites, in the intronic region of the *MC1R* gene, were identified in the hemp plumage breeds (n = 8), and no plausible SNPs were detected in these sites for the white chest black plumage breeds (n = 4) (Figure 5B).

## Discussion

Comparing with mallards and other domestic ducks, Jianchang ducks have great differences in morphology, physiology and behavior (Xiangdong *et al.* 2001) (Jiansheng 1987; Wang *et al.* 2018). Our current results basing on the population stratification analysis, showed that Jianchang ducks can be regarded as an independent branch from mallards and other domestic ducks. Our results are consistent with the findings basing on the analysis of estimate genetic diversity and genetic structure by microsatellite markers (Li *et al.* 2006). These analyses revealed different genetic backgrounds of Jianchang duck from other domesticated ducks and mallards, which reflect the genomic basis of unique characteristics of Jianchang duck.

All domesticated animals are originated from common ancestor wild animals (Li *et al.* 2010; vonHoldt and Driscoll 2016; Evin *et al.* 2017). We compared the genomic differences between mallard and jianchang duck, to check what genes have been selected during domestication process of jianchang duck. Based on 867 genes in the top 5% of *F*_ST_ and θπ region comparing between them, the GO terms were significantly enriched in energy metabolism and neural processes. Genes related to neural processes, such as *CNTN1*, *CHRNA9*, and *SHANK2*, showed particularly strong signals of selective sweeps presumably. *CNTN1* is involved in retinal axon regeneration, and *CHRNA9* affect inner ear development (Järvelä 2018). *CNTN1* and *SHANK2* are related to synapses formation and transmission regulation, which can affect memory by regulating neurotransmission (Haenisch *et al.* 2005; Lim *et al.* 2017; Monteiro and Feng 2017). The current study identified thirteen SNPs in the intron of *CNTN1*, and they were observed to have segregated between mallards and Jianchang ducks, suggesting *CNTN1* was under selection pressures during domestication of Jianchang duck. It is widely known that genes associated with neural processes were initially under selection pressures during the domestication of animals (Alberto *et al.* 2018; Lan *et al.* 2018; Zhang *et al.* 2018). Our findings were also consistent with the behavioral characteristics of animals at the primary stage of domestication. During this period, animals gradually adapted to a domesticated environment and became more docile and less responsive to environmental stimulation (Price 1984; Johnsson 2017). In fact, domestic ducks were less alert than mallards, and showed significant differences in brain morphology (Gille and Salomon 2000; Söderquist *et al.* 2017). Similarly with other domesticated ducks, in the early domestication process from mallards, the genes under big selection pressures in jianchang duck are also associated with neural processes.

Compared with other domestic duck breeds in China, Jianchang duck has the characteristics of fast growth rate and strong ability of lipid deposition in liver (Jiansheng 1987; Wang *et al.* 2018). The GO terms based on 370 enriched genes between Jianchang and other domestic populations were mainly enriched on cell growth and development, and lipid metabolism. Genes related to cell growth and development, and lipid metabolism have been enriched, and their functions have well investigated in growth development of organs and tissues, *e.g.*, PTPRJ (Balavenkatraman *et al.* 2006), *GLI2* (McCabe and Dattani 2014; Kramann *et al.* 2015; Kramann 2016), and *ESM1* (Yang *et al.* 2015). Particularly, *HSD17B12* was reported to have functions in the synthesis and metabolism of fatty acids (Engelen *et al.* 2012), and twelve SNPs identified in the intron of *HSD17B12* have segregated between Jianchang and other local breeds, suggesting the gene was under selection pressure and may contribute to fat deposition in Jianchang ducks.

*MITF* is an important gene locus with complex regulatory mechanisms, which are involved in pigmentation and melanocyte development, proliferation and survival in some vertebrates (Levy *et al.* 2006; Vachtenheim and Borovanský 2010). Zhou *et al.*, (2018) have shown that a novel intronic insertion most possibly leads to a splicing change in *MITF* accounted for white duck down feathers. Total nine SNPs distributed in the intron of *MITF*, have been identified to be segregated between colored plumage duck populations and white ones. Additionally, in the hair color study of quail, llamas, and pig (Wu *et al.* 2016; Kageyama *et al.* 2018; Lan *et al.* 2018), *MC1R* is related to the formation of brown hair. It has been well known that *MC1R* plays a central role in the regulation of eumelanin (black/brown) synthesis within the mammalian melanocyte (Kijas *et al.* 1998). By comparing the genomic variations of hemp plumage ducks and white breast black plumage ones, total nine SNPs located in the intron of *MC1R* were found to be differentiated, implying that *MC1R* is related to the hemp plumage of duck.

## Conclusion

We draw a conclusion that in the early domestication process of ducks, genes associated with neural processes were under selection pressures. Jianchang duck seems to have evolved earlier than other local breeds in China, and have genetic exchanges with other domestic and wild populations. The genes related to lipid metabolism were mainly selected and segregated from other local breeds, thus they may contribute to the characteristics of strong abilities in fat deposition in Jianchang ducks. *MITF* was identified to be related to white feather, and *MC1R* was related to hemp feather of Jianchang duck.
